# Lin28b Promotes Head and Neck Cancer Progression via Modulation of the Insulin-Like Growth Factor Survival Pathway

**DOI:** 10.18632/oncotarget.785

**Published:** 2012-12-29

**Authors:** Nehad M. Alajez, Wei Shi, Dennis Wong, Michelle Lenarduzzi, John Waldron, Ilan Weinreb, Fei-Fei Liu

**Affiliations:** ^1^ Stem Cell Unit, Department of Anatomy, College of Medicine, King Saud University, Riyadh, Saudi Arabia; ^2^ Ontario Cancer Institute, Toronto, Canada; ^3^ Department of Medical Biophysics, University of Toronto, Toronto, Canada; ^4^ Department of Radiation Oncology, University Health Network, Toronto, Canada; ^5^ Department of Radiation Oncology, University of Toronto, Toronto, Canada; ^6^ Department of Pathology, University Health Network, Toronto, Canada

**Keywords:** Lin28b, microRNA, Let-7, HNC, IGF

## Abstract

Lin28 is a developmentally regulated RNA binding protein which has recently emerged as key regulator in the biogenesis of the let-7 micro-RNA family. While the expression of Lin28b has been linked to advanced tumor stage, the precise molecular mechanism(s) by which Lin28b drives disease progression is still being unraveled. Herein, we generated a let-7-resistant Lin28b ORF, stably expressed in the FaDu head and neck cancer (HNC) cell line. FaDu-Lin28b cells exhibited enhanced tumor growth in vitro and in vivo. Global gene and micro-RNA expression analyses revealed significant enrichment in several pathways involved in cell migration, chromatin remodeling, and cellular stress response. Direct regulation of selected genes (HMGA2, CCND2, IGF1R, and IGF2BP2) via a let-7-Lin28b mechanism was validated. Notably, up-regulation of several genes in the IGF pathway in Lin28b-expressing cells was observed. Functional studies revealed significant increase in the survival of Lin28b-expressing cells when cultured under stress conditions, which was dependent on the presence of IGF1. Therefore, our data identified several novel gene targets for Lin28b-let7, and revealed a novel mechanism by which Lin28b promote tumorigenesis. Concordantly, clinical examinations of Lin28b, IGF2BP2 and IGF2 revealed a significant association between the expression of these genes with disease relapse, thereby corroborating the potential relevance for the Lin28b/IGF axis in HNC progression.

## INTRODUCTION

Lin28 is a developmentally regulated RNA binding protein which is expressed at high levels in undifferentiated embryonic stem cells (ESCs), but is dramatically reduced during differentiation [1]. Given its crucial role in maintaining the pluripotency of ESCs, this developmental regulator was subsequently found to be an integral member of the reprogramming gene set utilized to generate induced pluripotent stem cells (iPS)[2]. Shortly thereafter, Lin28 was also identified to function as a post-transcriptional regulator of Let-7 miRNA biogenesis, by inhibiting the microprocessor-mediated cleavage of pri-let-7 miRNAs [3]. Additionally, it could also induce uridylation of precursor let-7 (pre-let-7) at the 3' end, thereby inhibiting processing by DICER [4]. Furthermore, Lin28b-mediated poly-uridylation of pre-Let-7 miRNA was dependent on its interaction with the TUTase4 (TUT4) enzyme [5, 6]. *Lin28* over-expressing transgenic mice exhibited increased body size, crown-rump length, and delayed onset of puberty, which in turn were associated with increased glucose metabolism and insulin sensitivity [7]. In addition to its role in stem cell biology and development, high expression of Lin28 has also been associated with advanced stages in several different human malignancies [8, 9-12]. Not surprisingly, over-expression of Lin28 imparted radiation resistance properties mediated *via* K-ras regulation through a let-7 dependent mechanism [13]. Lin28 expression has also been linked to cancer stem cells [12, 14, 15].

Although the precise molecular mechanism(s) by which Lin28 drives tumorigenesis remains elusive, several cancer promoting genes (e.g. MYC, RAS, and HMGA2) have been reported to be direct targets for the let-7 miRNA family[16-18]. In this current study, we observed that over-expressing a let-7-resistant Lin28b gene in head and neck squamous cell carcinoma (HNSCC) was associated with enhanced tumour progression both *in vitro* and *in vivo*. Further investigations revealed significant suppression of Let-7 miRNA biogenesis in Lin28b-expressing tumor cells. Additionally, our study unravelled a novel molecular mechanism by which Lin28b promoted tumor survival under stress conditions through a let-7-IGF-dependent mechanism. Finally, in a cohort of 38 HNSCC patient tumour samples, over-expression of Lin28b, IGF2BP2, and IGF2 were observed to be associated with worse clinical outcome, strongly suggesting a role for the Lin28b-IGF pathway in contributing to HNSCC relapse.

## RESULTS

### Exogenous expression of Lin28b in FaDu cells promoted growth both *in vitro* and *in vivo*

**Figure 1:**
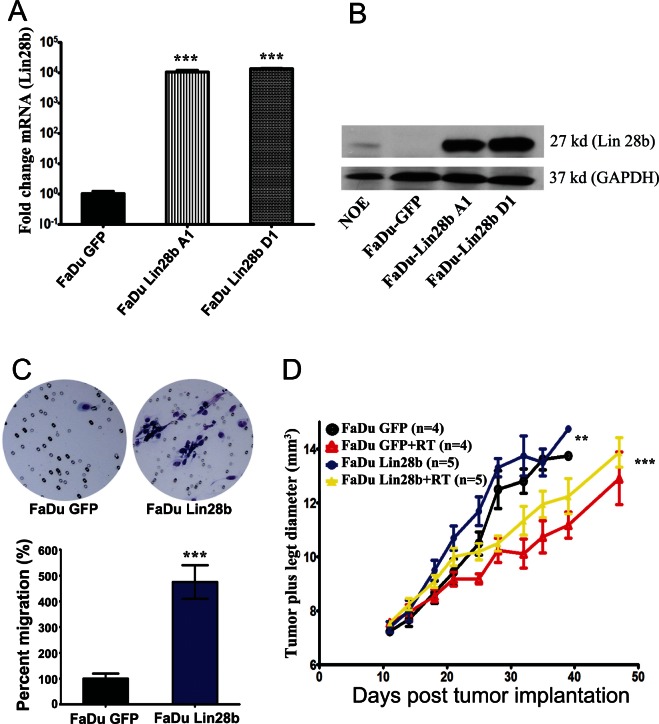
Stable expression of Lin28b enhanced HNSCC tumorigenicity *in vitro* and *in vivo* Expression of Lin28b in FaDu cells stably transfected with Lin28b-expression vector was detected by (A) qRT-PCR; and (B) Western blotting (27 kd), using GAPDH (37 kd) for loading control. (C) Representative images depicting enhanced migration ability of FaDu~Lin28b cells compared FaDu~GFP cells. Data were presented as mean ± S.E, n=10. (D) FaDu~Lin28b cells exhibited enhanced tumor formation and radiation resistance *in vivo*. Data were presented as mean ± S.E, n=4-5 mice/group. **p<0.005; ***p<0.0005.

Previous studies have reported significant association between Lin28b expression and advanced tumor stage; however, none of these studies examined the role of Lin28b in HNSCC [12-15]. Since Lin28b itself was subjected to Let-7 regulation, a Let-7-resistant Lin28b-expressing vector was constructed by cloning the Lin28b open reading frame (ORF), excluding the predicted Let-7 binding site in the 3' UTR region, into the pIRES-hrGFP II mammalian expression vector using the indicated primers in Supplementary Table 1. Stable transfection of the FaDu HNSCC tumor cell line (which has low endogenous Lin28b expression) with this plasmid led to a significant increase in Lin28b expression at both the mRNA and protein levels in two selected stable clones (A1 and D1), compared to GFP-transfected control cells (Fig 1A and B). Unless indicated otherwise, all subsequent experiments were conducted using the FaDu Lin28b D1 clone. Since advanced tumor stage often present with metastases, the migration capacity of Lin28b transfected FaDu cells *in vitro* was compared to that of parental control cells. As shown in Figure 1C, FaDu Lin28b cells exhibited a significantly higher migration potential compared to the GFP-transfected cells by ~5-fold. Furthermore, FaDu Lin28b expressing cells also exhibited enhanced proliferation *in vitro* (Supplementary Fig 1), plus increased tumour growth and radiation resistance *in vivo*, compared to the parental line (Fig 1D).

### Expression of Lin28b inhibited Let-7 miRNA biogenesis in FaDu Cells

**Figure 2:**
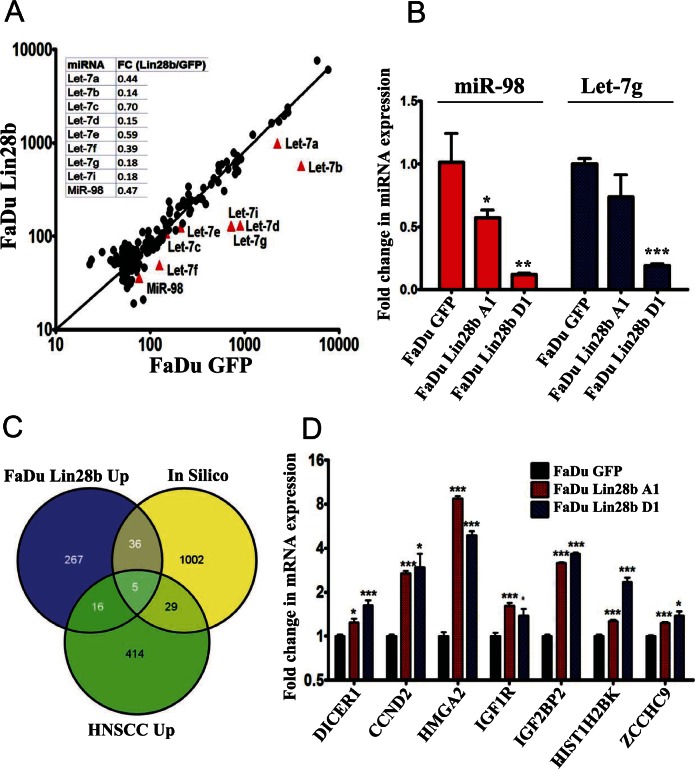
Characterizing the miRNA and mRNA changes in FaDu~Lin28b cells (A) FaDu~Lin28b or FaDu~GFP cells were subjected to global miRNA expression profiling using the nCounter NanoString platform, and the normalized expression value for each miRNA were plotted for the FaDu~GFP (x-axis) *vs*. the FaDu~Lin28b (y-axis) cells. (B) Expression of two representative let-7 miRNAs (miR-98 and Let7-g) was assessed in two stable FaDu~Lin28b clones (A1 and D1) using single-well Taqman miRNA assay. Data were presented as mean ± S.E, n=3. (C) Venn diagram depicting the intersection between the up regulated gene list in FaDu~Lin28b cells, *in silico* predicted Let-7 gene targets, and up regulated gene list from a publicly available microarray dataset (GSE6631) on primary HNSCC. (D) Validation of a selected group of genes identified from the microarray analyses. Data were presented as mean ± S.E, n=6. *p<0.05, **p<0.005, ***p<0.0005.

Lin28b has previously been shown to inhibit Let-7 miRNA biogenesis post-transcriptionally through interaction with TUT4, and by mediating pre-Let-7 polyuridylation [5]. To examine whether Let-7 micro-RNAs (miRNAs) were subjected to Lin28 regulation in this tumor model, the NanoString nCounter platform was utilized for global quantification of miRNA expression in FaDu~Lin28b compared to FaDu~GFP cells. The resulting data revealed significant down-regulation of several members of the Let-7 family in the FaDu~Lin28b cells, corroborating that Lin28b was indeed targeting this family of miRNAs in these cells (Fig 2A). The expression level of two of the down-regulated miRNAs (miR-98, and Let-7g) was validated in the FaDu~Lin28b cells using single-well quantitative RT-PCR (Fig 2B).

### Characterizing the downstream targets of Let-7 miRNAs in FaDu~Lin28b cells

In order to further examine the genes which were directly regulated by Let-7 miRNAs in FaDu~Lin28b cells, the same RNA preparation used for miRNA profiling was also subjected to global gene expression profiling using the Affymetrix HuGene-1_0-st-v1 array. Using 1.5-fold change as the cut-off, 324 genes were up-regulated in FaDu~Lin28b *vs*. FaDu~GFP cells (Fig 2C, FaDu Lin28b Up). Gene Ontology (GO) analysis on these 324 transcripts revealed significant enrichment in signal transduction, cell migration, and cellular response to insulin (Supplementary Table 2). These functions are consistent with the phenotype data of increased proliferation, migration and *in vivo* growth demonstrated in the FaDu~Lin28b cells (Fig 1C, 1D & Supplementary Fig 1). Since up-regulation of some of these genes in the FaDu~Lin28b cells could be induced indirectly or through a non-Let7-dependent mechanism, a non-redundant list of predicted Let-7 gene targets was compiled using the TargetScan miRNA target prediction database. When the up regulated gene list from the FaDu~Lin28b cells was crossed with the list of *in silico*-predicted Let-7 targets, there was an overlap in 41 genes (Fig 2C). To focus on this group of genes which should be more relevant to HNSCC biology, this up regulated gene list from the FaDu~lin28b cells, and the list of predicted Let-7 gene targets, was further cross-referenced with a third list of up-regulated genes in HNSCC identified by analyzing publicly available gene expression dataset from 22 HNSCC compared to 22 adjacent normal tissues (GSE6631). Using this strategy, 5 genes were identified to be common to all three aforementioned gene lists: HMGA2, CCND2, IGF1R, PAK1, and GNS. The expression level of 3 of these 5 overlapping, plus several additional genes (DICER1, CCND2, HMGA2, IGF1R, IGF2BP2, HIST1H2BK, and ZCCHC9), were validated in both FaDu~Lin28b clones “A1” and “D1” using qRT-PCR (Fig 2D).

### Direct regulation of HMGA2, CCND2, IGF2BP2, and IGF1R by Let-7 miRNA

**Figure 3:**
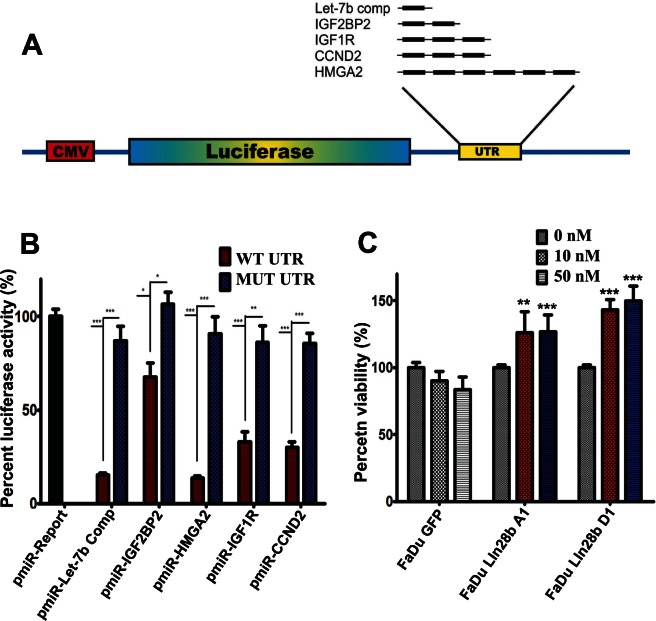
Activation of the IGF pathway in FaDu~Lin28b cells through a Let-7 dependent mechanism (A) Schema depicting the construction of luciferase reporter vectors carrying the indicated predicted let-7 miR binding sites downstream of the firefly *luciferase* gene in the pMIR-REPORT vector. The indicated number of Let-7 binding sites is shown in black bars. (B) The indicated wild type or mutant reporter vector was co-transfected with a pre-miR control (100 nM) or pre-miR Let-7b (100 nM) in HEK-293 cells, and luciferase activity was measured 24 hours later. Renilla luciferase activity was used for normalization. Data were presented as mean ± S.E, n=6. (C) FaDu~GFP, FaDu~Lin28b A1, and FaDu~Lin28b D1 cells were cultured in serum-free medium supplemented with 0, 10, or 50 ng/ml IGFI, and cell viability was measured on day 6 by MTS assay. Data were presented as mean ± S.E, n=6. *p<0.05, **p<0.005, ***p<0.0005.

The data thus far (Fig 2) suggested a direct regulation of several gene targets in FaDu~Lin28b cells *via* a Lin28b-Let-7 mechanism. To confirm that these genes were indeed targeted by Let-7 miRNAs, we constructed several reporter vectors carrying the predicted binding site(s) downstream of a firefly *luciferase* gene in the pMIR-Report vector as previously described [19] (Fig 3A). IGF2BP2 contains two predicted Let-7 binding sites; IGF1R and CCND2 each harbours three predicted Let-7 binding sites, while HMGA2 has six predicted Let-7 binding sites. For each construct, a mutant version of the reporter vectors in which the predicted Let-7 seed region(s) in the 3' UTRs was also generated using the primer combination listed in Supplementary Table 1. A positive and negative control reporter construct carrying either a wild type (wt) or (mut) Let-7b complementary sequence were also produced; pRL-SV40 (encoding for renilla luciferase) was used for normalization. Co-transfection experiments in HEK-293 cells demonstrated significant regulation of all tested constructs by Let-7b miRNA, which appeared to correlate with the number of binding sites for each construct (Fig 3B). The regulation of these UTRs by Let-7 was specific, as mutating the seed regions almost completely abrogated this effect.

It was noteworthy that several genes involved in the IGF pathway were up-regulated in the FaDu-Lin28b cells (IGF2BP2, IGF1R, IGFBP4, and IGF2BP3). These data hence suggested that activation of this pathway might be one mechanism by which Lin28b promoted tumor survival under stress conditions. This hypothesis was addressed by culturing FaDu-GFP, FaDu-Lin28b A1, and FaDu-Lin28b D1 cells under serum-free conditions (MEM-F15 without FBS), in the presence of differing concentrations of recombinant IGF1 (0, 10, or 50 nM). Consistent with our hypothesis, increased viability of Lin28b-expressing cells were indeed observed in the presence of IGF1, compared to control cells (Fig 3C), corroborating that survival of Lin28b cells were driven by a functionally intact IGF1 pathway.

### Expression of Lin28b, IGF2BP2, and IGF2 was associated with worse clinical outcome in HNSCC

**Figure 4:**
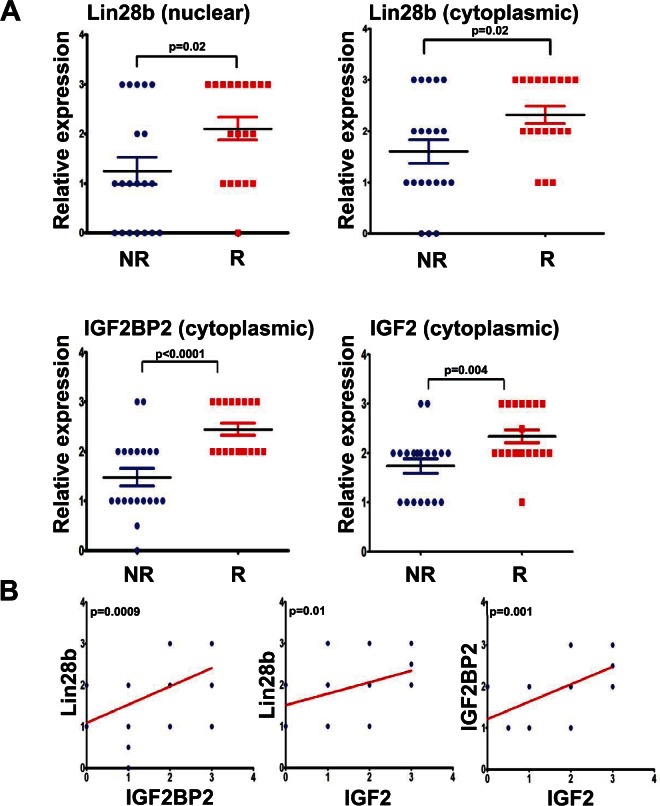
Expression of Lin28b, IGF2BP2, and IGF2 was associated with higher risk of recurrence in primary HNSCC samples (A) Expression of Lin28b (nuclear, or cytoplasmic), IGF2BP2 (cytoplasmic), and IGF2 (cytoplasmic) was assessed in 39 HNSCC samples by immunohistochemistry. The relative expression level of each protein was plotted as a function of patient outcome: recurrent (R); non-recurrent (NR). (B) Positive correlations were observed between Lin28b with IGF2BP2 and IGF2, as well as between IGF2BP2 with IGF2 expression in the same cohort of HNSCC specimens as presented in A.

Given that Lin28b-expressing cells were associated with increased cellular proliferation, migration, tumour formation, and radiation resistance, which appeared to be mediated through activation of the IGF pathway, we sought to determine whether Lin28b, IGF2BP2, and IGF2, bore any relationship to clinical outcome in HNSCC patients. To that end, expression levels of these three proteins was examined in the diagnostic formalin-fixed paraffin-embedded (FFPE) tumour biopsies in a selected cohort of 39 HNSCC patients wherein 19 patients had experienced a relapse; 20 have not, using immunohistochemistry (IHC). The clinical information on these patients is provided in Table 1, demonstrating that these two groups (non-relapsed *vs*. relapsed) were matched for important clinical parameters such as age, gender, tumour site, stage, and treatment. Representative immunostaining of HNSCC tissues by the aforementioned antibodies is shown in Supplementary Figure 2. The resulting IHC data demonstrated a significant association between higher Lin28b (cytoplasmic and nuclear), IGF2BP2 (cytoplasmic), and IGF2 (cytoplasmic) expression with relapse in this cohort of HNSCC patients (Fig 4A). Furthermore, a significant correlation was also observed between Lin28b with both IGF2BP2 and IGF2 expression, as well as between IGF2BP2 with IGF2 expression in these HNSCC specimens (Fig 4B), strongly suggesting that the regulation of these proteins might indeed be mediated through Lin28b.

**Table 1:** Clinical Characteristics of 39 HNSCC Patients

Factor	Total(n=39)	Non-recurrence(n=20)	Recurrence(n=19)
Age(Median, Range)	67(50-92)	70(50-92)	63(50-87)
Gender(M/F)	31/8	16/4	14/5
Tumor site			
-Larynx	28	17	11
-Hypopharynx	11	3	8
Stage			
III	16	8	8
IVA	22	12	10
IVB	1	0	1
Treatment Intent			
RT	26	14	12
CRT	13	5	8

RT(Radiotherapy); CRT(Chemo-Radiotherapy)

## DISCUSSION

There is compelling evidence that miRNAs play important roles in mediating tumor development and progression, primarily through the regulation of their respective target genes [8, 19-34]. Adding to this complexity however, is the emerging data demonstrating that miRNAs themselves are subjected to post-transcriptional regulation by other genes. One of the best characterized examples is the negative feedback loop between the Let-7 miRNA family and Lin28, wherein Lin28 regulated Let-7 miRNA family biogenesis through interaction with TUT4 and polyuridylation [5]; and in turn, Lin28 itself is also subjected to Let-7 miRNA regulation [35]. In the current study, we observed significant down-regulation of almost the entire let-7 miRNA family in stably-transfected FaDu~Lin28b cells (Fig 2A and B). Our groups had previously profiled primary HNSCC samples, and reported the down regulation of several members of the Let-7 miRNA family, although the molecular mechanisms leading to Let-7 under-expression at that time was not elucidated [36]. Thus, our current data provide one biological explanation for the Let-7 miRNA down regulation in HNSCC, mediated through Lin28b. While Lin28b has been reported to promote tumour progression, the precise mechanism(s) thereof has remained unclear. Previous reports have described Lin28 down-regulation of the Let-7 family, which in turn led to the increased expression of target oncogenes such as HMGA2 and RAS [17, 18]. Our data similarly identified HMGA2 up-regulation in Lin28b over-expressing cells, consistent with these previous observations.

**Figure 5:**
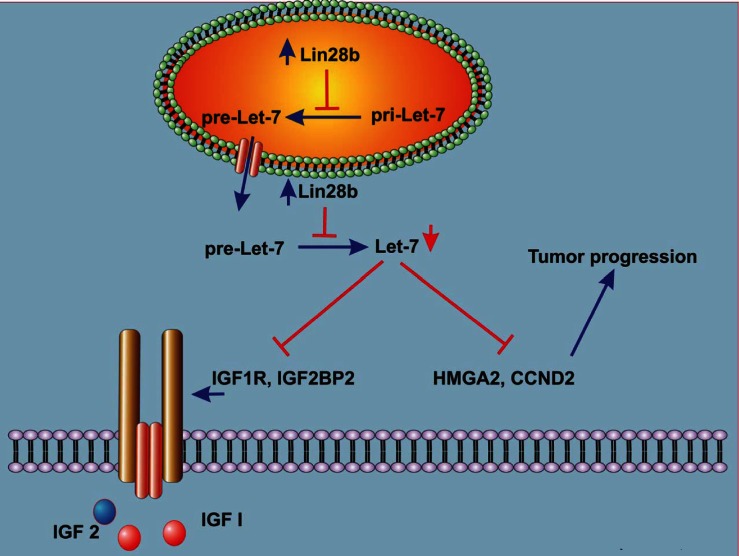
Schema describing a model for Lin28b-mediated tumor progression Over-expression of Lin28b in HNSCC cells inhibits let-7 miRNA family biogenesis. In turn, down-regulation of mature Let-7 miRNAs in Lin28b-expressing cells allows the over-expression of several Let-7 gene targets with known roles in tumor progression, such as HMGA2, and CCND2. Furthermore, down-regulation of Let-7 miRNAs in Lin28b-expressing cells also leads to up regulation of several genes in the IGF pathway such as IGF1R, and IGF2BP2, which promotes tumor survival under stress conditions.

The novelty of our current study however, relate to the identification of additional Let-7 gene targets in Lin28b over-expressing cells, with CCND2, IGF2BP2, and IGF1R being amongst the most prominent targets, experimentally corroborated by the luciferase reporter system. Furthermore, our data have unravelled yet another mechanisms by which Lin28b could promote tumour progression, namely through activation of the IGF pro-survival pathway (Fig 5). Previous reports have demonstrated regulation of IGF1R *via* Lin28 and Let-7 mechanisms in Lin28a transgenic mice, associated with increased body size, crown-rump length and delayed onset of puberty [7]. To the best of our knowledge, this is the first report linking regulation of the IGF pathway by the Lin28/Let-7 axis to human cancer progression. Furthermore, we demonstrated that Lin28b expressing cells were more responsive to IGF stimulation under serum-free conditions, strongly implicating a role for Lin28b in enhancing tumour survival under stress conditions. The biological and clinical relevance of this axis was corroborated in primary HNSCC samples, wherein over-expression of these three proteins along this axis: Lin28b, IGF2BP2, and IGF2 were all significantly associated with a higher risk of relapse, highlighting the importance of this pathway in driving HNSCC progression.

The potential relevance of the IGF pathway in HNSCC has long been noted, with many opportunities for therapeutic targeting [37]. Pre-clinical evidence certainly supports this strategy [38], although a recently published Phase II clinical trial in metastatic HNSCC patients using an IGF-1R monoclonal antibody as mono-therapy has proven to be disappointing [39]. Given the complexity of this pathway, with multiple ligand and receptor interactions in epithelial cancer cells, the interplay between the stroma with the malignant epithelial tissues, along with the interactions between the IGF and IGFBPs, it would have been indeed quite surprising if a single molecular therapy would have been therapeutically effective, delivered as the sole modality. Nonetheless, this current work provides additional biological insights into yet another mechanism by which this complex pathway can be activated in HNSCC.

In conclusion, we have demonstrated a novel mechanism by which Lin28b over-expression promotes tumour progression *via* additional Let-7 gene targets such as CCND2, IGF2BP2, and IGF1R. In turn, over-expression of Lin28b, IGF2BP2, and IGF2 in patients’ tumours at the time of diagnosis was all associated with a higher risk of relapse. Collectively, this underscores the biological importance and relevance of this complex pathway, further supporting the continued interrogation of this axis in HNSCC progression.

## METHODS

### Ethics statement

All tissue studies have received University Health Network institutional REB approval. All animal experiments were conducted in accordance with the guidelines of the Animal Care Committee, University Health Network.

### Patients

The clinical information for the patients’ tissues used in this study has been summarized in Table 1. Patients had either laryngeal, or hypopharyngeal squamous cell carcinomas, treated for cure, with either radical radiation therapy (RT) alone, or combined with chemotherapy (Cisplatin). The median follow-up for this cohort of 39 patients was 2.8 years. The recurrent *vs*. non-recurrent cases were selected to be matched for age, gender, tumour site, stage, and treatment.

### Cell culture

Human normal oral epithelial cells (NOE) were obtained from Celprogen (San Pedro, CA), and were cultured as previously described [40]. The HNSCC FaDu cell line was obtained from American Type Culture Collection (Manassas, VA). Cells were cultured in MEM F-15 supplemented with 10% fetal bovine serum, 100 mg/l penicillin, and 100 mg/l streptomycin at 37°C and 5% CO_2._ FaDu-GFP, and FaDu-Lin28b hypopharyngeal tumor cell lines were maintained under similar culture conditions.

### Cloning of a Let-7 resistant Lin28b ORF expression vector

The full length Lin28b ORF (lacking the 3' UTR and the predicted Let-7 binding sites) was amplified using the indicated primes in Supplementary Table 1, and the SuperScript III One-Step RT-PCR System with Platinum *Taq* High Fidelity kit (Invitrogen). Subsequently, the PCR product was gel purified and ligated into the pcDNA3.1 TOPO vector according to the manufacture's recommendations (Invitrogen, CA). Subsequently, Lin28b was sub-cloned into the *BamH I* and *Not I* sites in the pIRES-hrGFP II mammalian expression vector (Agilent Technologies, Santa Clara, CA, USA). Stable clones were then generated by transfecting FaDu cells with Lin28b expression vector, followed by selection in G418 (1mg /ml), and FACSorting.

### Cell viability assay

Cell viability was assessed using the soluble tetrazolium salt (MTS) cell proliferation assay as described previously[20]. For serum-free culture experiments, 5000 FaDu, FaDu~GFP, or FaDu~Lin28b cells were cultured in MEM-F15 containing no serum, and supplemented with the indicated concentrations of recombinant IGFI (R&D Systems, Minneapolis, MN,USA). At the indicated time points, 20 ul of the MTS reagent (Promega, Madison, WI, USA) were added to each well in a 96-well plate, and absorbance was measured at *λ*_492_.

### Cell migration

Cell migration was assessed as also previously described [19], using the 6-well BD trans-well migration system (BD Biosciences, Franklin Lakes, NJ). Three ml RPMI supplemented with 15% FBS was added to the lower chamber, while 3'10^5^ FaDu~GFP, FaDu~Lin28b cells were re-suspended in RPMI plus 1% FBS, added to the upper chamber (2 mls). Twenty-four hours later, inserts were fixed and stained using SIEMENNS DIFF-QUICK stain set (Siemens Healthcare Diagnostics, Deerfield, IL), and the number of migrating cells in five 10x fields was then quantified.

### Micro-RNA and gene expression profiling

Global micro-RNA expression profiling of FaDu~Lin28b and FaDu~GFP cells was conducted using the NanoString nCounter miRNA system, while gene expression profiling of the same RNA preparation was conducted using the Affymetrix HuGene-1_0-st-v1 array, all conducted at the University Health Network Microarray Center (Toronto, Canada). Gene expression data were normalized using R (version 2.7.1) software and the aroma.affymetrix package as described before [20]. A candidate Let-7 family gene target list was generated by compiling a non-redundant list of *in silico* predicted targets using the TargetScan miRNA target prediction database. Gene Ontology (GO) and pathway analyses on Let-7 targets were conducted using DAVID Bioinformatics Database functional-annotation tools, as we have previously described [19, 41].

### qRT-PCR for mRNA and miRNA expression

QRT-PCR analyses were performed using the ABI PRISM 7900 Sequence Detection System (Applied Biosystems Inc., Foster City, CA, USA). The primer sequences used in this study are listed in Supplementary Table 1. Total RNA was isolated using the Norgen total RNA purification kit (Norgen Biotek Corporation, Canada). Reverse transcription was performed using SuperScript III Reverse Transcriptase (Invitrogen Corp.) according to the manufacturer's recommendations. The relative fold change in RNA expression was calculated using the 2^βΔΔCt^ method, where the average of Δ*C*t values for the amplicon of interest was normalized to that of an endogenous gene (GAPDH), compared with control specimens. Single-well miRNA PCR were conducted using Taqman MicroRNA Assay (Applied Biosystems), as we have previously reported [19].

### Immunoblotting

Immunoblotting was done as described before [42]. NOE, FaDu, FaDu~Lin28bA1, and FaDu~Lin28b D1 cells were collected and lysed in 1 M Tris-HCl (pH 8), 5 M NaCl, and 1% NP40 plus protease inhibitor cocktail (Roche Diagnostics, Quebec, Canada). Protein concentration was determined using the Bio-Rad Detergent-Compatible Protein Assay (Bio-Rad Laboratories, Hercules, CA, USA). In total, 20 ug of protein were loaded onto 10% Tris-glycine protein gels (Invitrogen) for electrophoresis. The protein was then transferred onto a nitrocellulose membrane using a Trans-Blot SD Semi-Dry Transfer Cell (Bio-Rad) and blocked using TBST (0.1% Tween-20 plus 5% fat-free dry milk). The membrane was probed with anti-Lin28b (1:100 dilution; Abcam# ab71415), and anti-GAPDH (Abcam, Cambridge, MA, USA) antibodies, as previously described [41].

### Luciferase Reporter Assay

A reporter vector (pMir-Report, ABI) carrying the predicted Let-7 binding site(s) from each 3' UTR was constructed using partially complementary primer pairs as indicated in Supplementary Table 1 and Figure 3, using Amplitaq gold DNA polymerase (Applied Biosystems Inc, Foster City, CA). IGF2BP2 has two putative Let-7 binding sites; IGF1R and CCND2 each have three putative binding sites, while HMGA2 has six putative binding sites. As positive control, we constructed a vector carrying the full length complementary sequence to Let-7b miRNA. A mutant version of each reporter plasmid was generated by mutating the seed region for the let-7 miRNA family using the indicated primers in Supplementary Table 1. All regions were subsequently cloned into the SPE I and HIND III sites downstream of the firefly *luciferase* gene in the pMIR-REPORT vector (Applied Biosystems Inc, Foster City, CA). To assess the direct interaction between Let-7 miRNA family and the 3'UTR from the aforementioned targets, HEK-293 cells were transfected with 100 nM of pre-miR-ctrl, or pre-miR-Let-7b. Six hours later, cells were co-transfected with 100 ng of pMIR-REPORT carrying either wt or mutant 3' UTR sequences, along with 20 ng of pRL-SV40 vector (Promega) carrying the *Renilla luciferase* gene. Transfection experiments were conducted using Lipofectamine 2000 (Invitrogen). At 48 hrs post-transfection, luciferase activity was measured using the Dual-Glo luciferase assay system (Promega). Firefly luciferase activity was then normalized to that of *Renilla* luciferase.

### *In vivo* experiments

For tumor formation assay, 5'10^5^ FaDu~GFP or FaDu~Lin28b cells were injected intramuscular (IM) into the left gastrocnemius muscle of 6–8-week-old female SCID mice. Once tumours started to grow, half of the tumor-bearing mice were exposed to 5 Gy local RT as described before [43]. Briefly, mice were immobilized in a Lucite box and the tumor-bearing leg was exposed to 225 kV (13 mA) at a dose rate of 3.37 Gy/min (X-RAD 225C Biological X-Ray Irradiator; Precision X-Ray, North Branford, CT). Tumor growth was monitored by measuring tumor plus leg diameter as described previously [41].

### Immunohistochemistry

Lin28b, IGF2BP2, and IGF2 immunohistochemistry evaluations were performed on 4-um FFPE tumor sections using microwave antigen retrieval, in combination with the LSAB+System-HRP (Dako North America, Carpinteria, CA, USA). Detection of these proteins utilized rabbit anti-Lin28b (1:50 dilution; Abcam# ab71415), anti-IGF2BP2 (1:500 dilution, Novus Biological), and anti-IGF2 (1:250 dilution, Abcam# ab9574) antibodies. Expression was scored as previously described [20]. Briefly, for cytoplasmic staining, the intensity of tumor cells staining was evaluated as 0, 1, 2 and 3. Tumor nuclear staining was also calculated on 3 staining fields with at least 300 tumor cells, as 0, 1: <25%, 2: 25-50%; 3: >50%.

### Statistical analysis

Statistical analyses and graphing were performed using Microsoft excel 2007 and Graphpad Prism 5.0 software (Graphpad software, San Diego, CA, USA).

## Supplementary Figures and Tables




