# Supplementary material for: Lin28b Promotes Head and Neck Cancer Progression via Modulation of the Insulin-Like Growth Factor Survival Pathway
Source: Oncotarget. 2012 Dec 29;3(12):1641–52. doi: 10.18632/oncotarget.785 (PMC3681501; doi:10.18632/oncotarget.785)
Supplement: Supplementary file 1 [file oncotarget-03-1641-s001.pdf]

Lin28b Promotes Head and Neck Cancer Progression via Modulation of the Insulin-Like Growth Factor Survival Pathway – Alajez et al

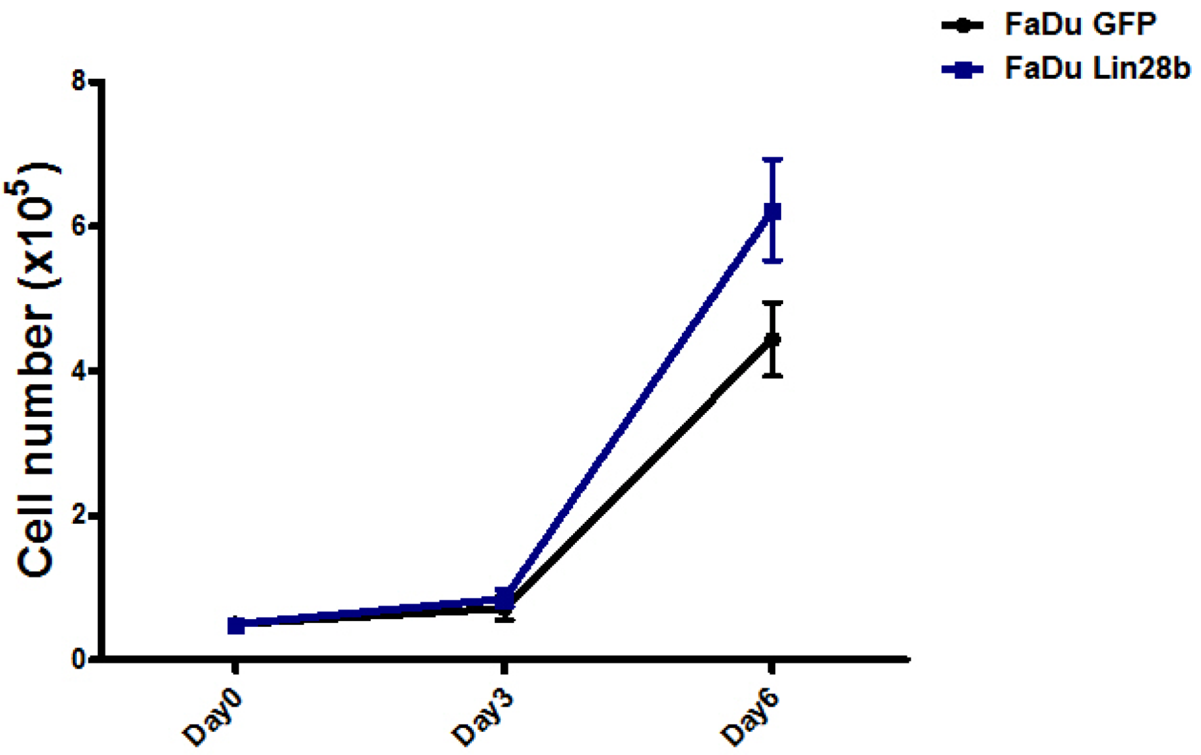

Supplementary Figure 1. Lin28b enhanced tumor cell proliferation in vitro. FaDu Cells stably transfected with GFP (blue square) or with Lin28b (black circle) were cultured in DMEM+10% FBS, and total cell number was counted on days 0, 3, and 6. Data are presented as mean ± SD, n=3.

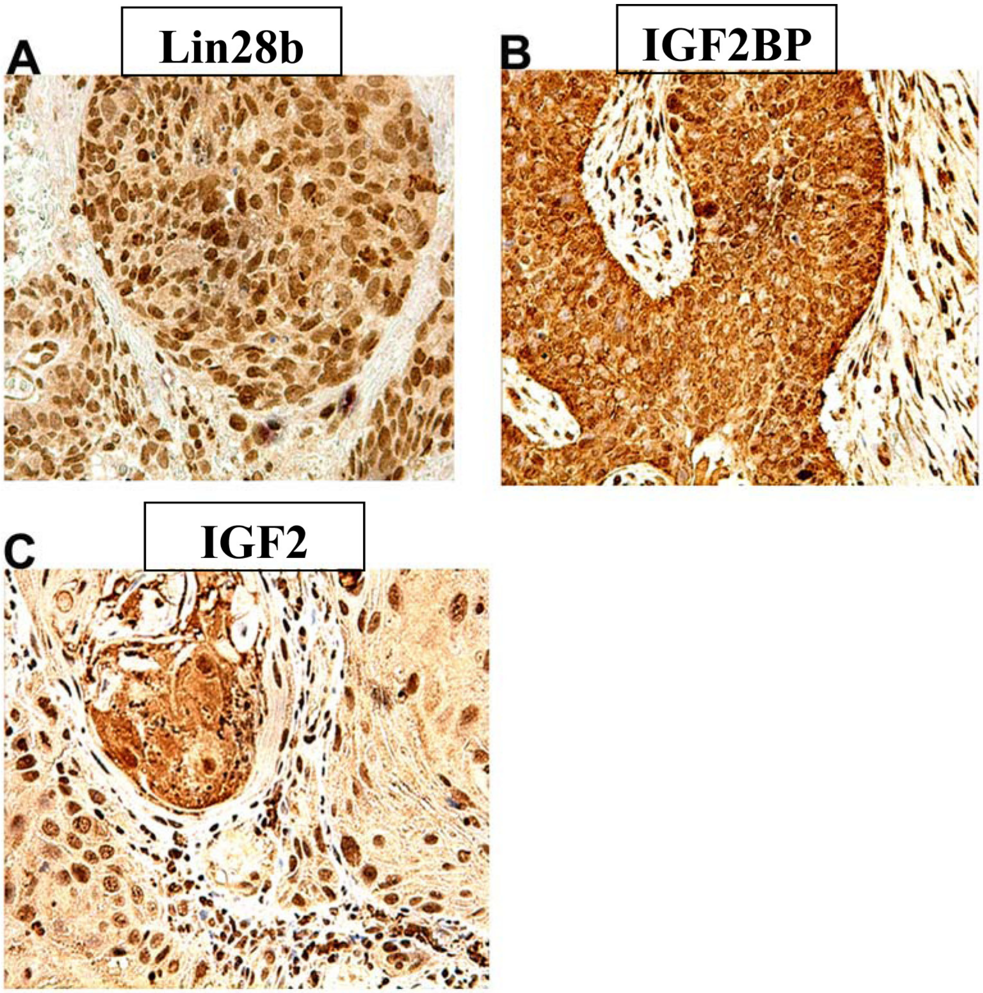

Supplementary Figure 2: Representative Immunohistochemistry staining for Lin28b (nuclear and cytoplasmic, A), IGF2BP2 (cytoplasmic, B), and IGF2 (cytoplasmic, C) in representative HNSCC FFPE sections.
